# Photocatalytic Partial Water Oxidation Promoted by a Hydrogen Acceptor‐Hydroxyl Mediator Couple

**DOI:** 10.1002/advs.202410680

**Published:** 2024-12-20

**Authors:** Yuanqiang Mai, Dongsheng Zhang, Kristina Maliutina, Xueyang Leng, Nengjun Cai, Jialu Li, Chao Wang, Yu Huang, Kai Zhang, Wujun Zhang, Yongwang Li, Flemming Besenbacher, Hans Niemantsverdriet, Wenting Liang, Yanbin Shen, Tingbin Lim, Emma Richards, Ren Su

**Affiliations:** ^1^ Soochow Institute for Energy and Materials InnovationS (SIEMIS) Soochow University Suzhou 215006 China; ^2^ SynCat@Beijing Synfuels China Technology Co. Ltd. Leyuan South Street II, No.1, Yanqi Economic Development Zone C# Beijing 101407 China; ^3^ School of Chemistry Cardiff University Park Place Cardiff CF10 3AT UK; ^4^ Institute of Environmental Science School of Chemistry and Chemical Engineering Shanxi University Taiyuan 030006 China; ^5^ Suzhou Institute of Nano‐Tech and Nano‐Bionics (SINANO) Suzhou Industrial Park Suzhou 215123 China; ^6^ State Key Laboratory of Coal Conversion Institute of Coal Chemistry CAS Taiyuan 030001 China; ^7^ Interdisciplinary Nanoscience Center Aarhus University Gustav Wieds Vej 14 Aarhus DK‐8000 Denmark; ^8^ Syngaschem BV Valeriaanlaan 16 Nuenen 5672 XD The Netherlands; ^9^ Joint School of National University of Singapore and Tianjin University International Campus of Tianjin University Bin‐hai New City Fuzhou 350207 China

**Keywords:** heterogeneous photocatalysis, hydrogen acceptor, hydrogen peroxide, OH mediator, water oxidation

## Abstract

Hydrogen peroxide (H_2_O_2_) is an important chemical in synthetic chemistry with huge demands. Photocatalytic synthesis of H_2_O_2_ via oxygen reduction and water oxidation reactions (ORR and WOR) is considered as a promising and desirable solution for on‐site applications. However, the efficiency of such a process is low due to the poor solubility of molecular oxygen and the rapid reverse reaction of hydroxyl radicals (^•^OH) with hydrogen atoms (H). Here, a strategy is proposed to boost the H_2_O_2_ evolution via oxidation of water by employing a H acceptor (**A**, nitrocyclohexane), an ^•^OH mediator (**M**, dioxane), and a photocatalyst (CdS nanosheets). While ^•^OH radicals are stabilized by dioxane to produce ketyl radicals prior to the formation of H_2_O_2_, H atoms are effectively utilized in the generation of cyclohexanone oxime, an important intermediate in the production of Nylon 6. The system displays a rapid kinetic accumulation of H_2_O_2_ (0.13 min^−1^) to a high concentration (6.6 mm). At optimum reaction conditions, a high quantum efficiency (16.6%) and light‐to‐chemical conversion efficiency (4.9%) can be achieved under 410 nm irradiation.

## Graphical abstract

Photocatalytic partial water oxidation promoted by a hydrogen acceptor (**A**) and a hydroxyl mediator (**M**) couple on CdS nanosheets. While the photogenerated ^•^OH radicals can be stabilized by **M** to produce ketyl radicals prior to the formation of H_2_O_2_, H atoms are transferred to **A**. This strategy enables a rapid evolution of H_2_O_2_ with a high quantum efficiency (16.6%) under visible light irradiation.

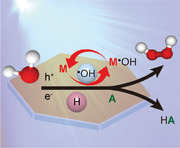

## 1 Introduction

Hydrogen peroxide (H_2_O_2_) is an eco‐friendly oxidant that is widely used in synthetic chemistry, in bleaching and disinfecting, and degradation of pollutants.^[^ ^1^ ^]^ It is also considered as a promising energy carrier and is among the “100 most important chemical compounds”.^[^ ^2^ ^]^ The global annual production of H_2_O_2_ is ∼4 million tons,^[^ ^3^ ^]^ ∼95% of which is produced via the anthraquinone (AQ) cycling process.^[^ ^4^ ^]^ This process involves the hydrogenation of AQ, oxidation of hydroanthraquinone (AQH), extraction of H_2_O_2_, and recovery of active quinones from deteriorated molecules, rendering it suitable for production at large scale, but impractical for on‐site applications (i.e., disinfection, synthesis) at smaller volumes. Additionally, poisoning of the supported Pd catalyst (employed in the initial hydrogenation step), limitations in mass transfer and solubility of oxygen, and over‐hydrogenation of AQ need to be considered.^[^ ^5^ ^]^ It is therefore essential to develop a sustainable and efficient method for on‐site H_2_O_2_ production, ideally employing H_2_O and O_2_ via photocatalysis and/or electrocatalysis under ambient reaction conditions.^[^ ^6^ ^]^

Photocatalytic synthesis of H_2_O_2_ via the oxygen reduction and water oxidation reactions (ORR and WOR) has attracted massive attention and a series of engineered photocatalysts has been developed, including inorganic and polymeric semiconductors,^[^ ^7^ ^]^ covalent organic frameworks (COFs),^[^ ^8^ ^]^ and metal–organic frameworks (MOFs).^[^ ^9^ ^]^ The 2e^−^ ORR path is the most investigated approach for the synthesis of H_2_O_2_ owing to a relatively low redox potential (0.695 V vs standard hydrogen electrode, SHE).^[^ ^1^ ^]^ Teng et al. show that the adsorption of O_2_ can be manipulated by loading single Sb atoms on graphitic carbon nitride to promote the formation of Sb‐μ‐peroxide (Sb‐OOH) intermediates, leading to an efficient evolution of H_2_O_2_ via the ORR pathway under visible light.^[^ ^7c^ ^]^ Gopakumar et al. demonstrate that lignin functionalized BiOBr displays a suitable redox potential for the activation of molecular oxygen in seawater.^[^ ^7a^ ^]^ Interestingly, the functional groups of the lignin also serve as an electron sink for the ionization of metal cations, thus further enhancing the evolution of H_2_O_2_. In order to achieve satisfactory generation rates of H_2_O_2_, sacrificial agents (e.g., isopropanol and ethanol) with fast oxidation kinetics are often added into the system.^[^ ^9^ ^]^ Note that the generated active oxygen species (e.g., ^•^OOH) may decompose or react with the sacrificial agents, thus reducing the efficiency for the generation of H_2_O_2_. Additionally, pure oxygen is sometimes employed to improve the concentration of molecular oxygen in the liquid, which poses risks for practical applications.^[^ ^7h^ ^]^ H_2_O_2_ can also be produced from the partial water oxidation reaction (WOR) via a direct 2e^‒^ process or an indirect formation of hydroxyl radical intermediates (^•^OH), which is often observed in heterogeneous photocatalysis.^[^ ^7^, ^8^ ^]^ The concerns regarding the solubility of oxygen and hole scavengers are thus eliminated. Ren et al. show that a reasonable evolution of H_2_O_2_ from WOR can be realized by employing atomically dispersed Mn supported on an aryl amino‐substituted g‐C_3_N_4_ photocatalyst under visible light irradiation (∼3.2 mm for 7 h).^[^ ^7l^ ^]^ Though the redox potential of H_2_O/H_2_O_2_ (1.77 V vs SHE) is relatively lower than H_2_O/^•^OH (2.7 V vs SHE), the catalytic performance is limited by the inherent slow kinetics of the 2e^−^ process.^[^ ^10^ ^]^ In comparison, the efficiency of the indirect process is severely limited by the rapid reverse reaction of the photogenerated ^•^OH radicals and H atoms.^[^ ^7l,m^ ^]^

**Scheme 1:**
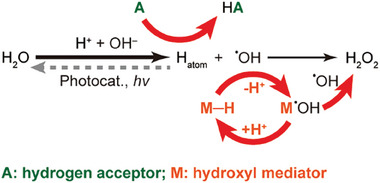
The strategy. Photocatalytic H_2_O_2_ formation via WOR with a hydrogen acceptor and hydroxyl mediator couple.

The overall efficiency for H_2_O_2_ evolution from oxidation of ionized water in a basic media (OH^‒^/^•^OH, 1.99 V vs SHE) may be improved by introducing a hydrogen acceptor (**A**) and a hydroxyl mediator (**M**) pair, as proposed in **Scheme** **1**. The depletion of photogenerated active hydrogen species by a hydrogenation reaction would prolong the lifetime of the ^•^OH, which interacts with **M** to yield H_2_O_2_ via the formation of a metastable **M** ^•^OH adduct, which reduces back to **M** in a complete cycle. This not only inhibits the unwanted recombination of ^•^OH with H atoms, but also offers an opportunity for the synthesis of valuable hydrogenation products when a suitable **A** is used. The procedure also calls for a photocatalyst with appropriate bandgap and band positions to realize fast transfer rates of H atoms and ^•^OH radicals. Additionally, for practical considerations, **A**, **M**, and H**A** should be stable at a relatively high concentration of H_2_O_2_.

Here we show that efficient synthesis of H_2_O_2_ via the WOR pathway can be realized by employing nitrocyclohexane, dioxane, and CdS nanosheet as **A**, **M**, and photocatalyst, respectively, under visible light irradiation. Photogenerated hydrogen atoms hydrogenate nitrocyclohexane into cyclohexanone oxime, an important intermediate in the production of Nylon 6, while ^•^OH radicals rapidly react with dioxane to produce the corresponding ketyl radical adduct prior to the formation of H_2_O_2_. The CdS nanosheets display appropriate band positions for both valence band (VB, 2.0 V vs SHE) and conduction band (CB, −0.7 V vs SHE) and provide excellent charge separation efficiency to drive both half reactions, resulting in a rapid accumulation of H_2_O_2_ (13.2 mm h^−1^) to a high concentration (6.6 mM). Remarkably, a high quantum efficiency (QE) of 16.6% and light‐to‐chemical conversion efficiency (LCC) of 4.9% are achieved under 410 nm irradiation, producing 0.13 mmol H_2_O_2_ within 30 min.

## 2 Results and Discussion

### 2.1 The Photocatalyst

**Figure 1:**
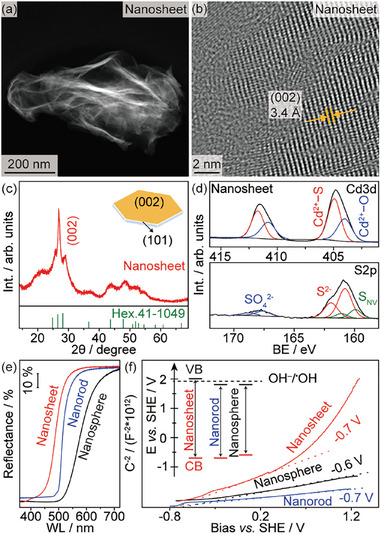
Characterizations of the photocatalysts. a–d) TEM images, XRD, and XPS of the CdS nanosheets. e,f) DRS and Mott–Schottky plots of CdS nanosheets in comparison with nanorods and nanospheres. Inset: Derived band positions.

CdS photocatalysts with controllable shapes (nanosheet, nanorod, and nanosphere) were synthesized by a solvothermal method using Cd(OAc)_2_∙2H_2_O and CH_4_N_2_S as precursors (Note S1, Supporting Information).^[^ ^11^ ^]^ Transmission electron microscopic imaging (TEM) reveals the successful synthesis of shaped CdS (**Figures** **1**a; Figure S1, Supporting Information). The CdS nanosheets are characterized by a lattice spacing of 3.4 Å that corresponds well with the presence of (002) facets (Figure 1b). X‐ray diffraction shows a sharp (002) diffraction peak for the CdS nanosheet photocatalyst (XRD, Figure 1c; Figures S2 and S3, Supporting Information), confirming the plate‐like structure, agreeing well with the HRTEM analysis. According to previous investigations,^[^ ^12^ ^]^ photogenerated electrons tend to migrate to the (002) facet of the CdS nanosheet, which facilitates spatial charge separation, thus benefiting the overall efficiency of photocatalytic reactions.

X‐ray photoelectron spectroscopy (XPS) reveals that all CdS photocatalysts consist of Cd, S, O, and adventitious C species (Figure S4, Supporting Information). Noticeably, the Cd3d spectra indicate that the surface of the CdS nanosheets contains Cd^2+^−S (404.9 eV) and Cd^2+^−O (404.0 eV) species (Figure 1d),^[^ ^13^ ^]^ possibly caused by the leaching of S^2‒^ from the (002) facet during the solvothermal synthesis. This is evidenced by the S2p spectra of the CdS nanosheets (Figure 1d), where the vacancy‐neighboring S atom (S_NV_, 159.9 eV) and SO_4_^2‒^ (167.6 eV) species are observed along with lattice S^2‒^ species (160.8 eV).^[^ ^14^ ^]^ In comparison, the CdS nanorods and nanospheres only show characteristic Cd^2+^ and S^2‒^ species (Figures S2 and S3, Supporting Information). The vacancy‐rich (002) facets of the CdS nanosheets provide undercoordinated surface Cd species that benefit the storage and interfacial charge transfer of photogenerated electrons for reduction reactions.^[^ ^12^, ^15^ ^]^ The optical bandgap of the CdS nanosheets is determined to be ∼2.7 eV according to the diffuse reflectance spectrum (DRS), which is slightly larger than the nanorods (2.5 eV) and nanospheres (2.4 eV, Figure 1e). Since the flat‐band potentials (V_Fb_) for all three CdS photocatalysts are similar (∼−0.6 to −0.7 V vs SHE) according to the Mott–Schottky plots (Figure 1f), the VB of the CdS nanosheets (2.0 V) is more positive than those of the CdS nanorods and nanospheres (both 1.8 V, inset of Figure 1f). This agrees well with the estimated VB derived from ultra‐violet photoelectron spectroscopy (UPS, Figure S5, Supporting Information). Apparently, the VB and CB of the CdS nanosheets match well with the redox potentials of OH^‒^/^•^OH and a few hydrogenation reactions,^[^ ^16^ ^]^ and thus are thermodynamically favorable for the complete reaction. Additionally, the CdS nanosheets display a relatively large specific surface area (94.3 m^2^ g^−1^) in comparison to the nanorods (21.7 m^2^ g^−1^) and nanospheres (8.1 m^2^ g^−1^) according to N_2_ adsorption isotherms (Figure S6, Supporting Information).

### 2.2 Performances

**Figure 2:**
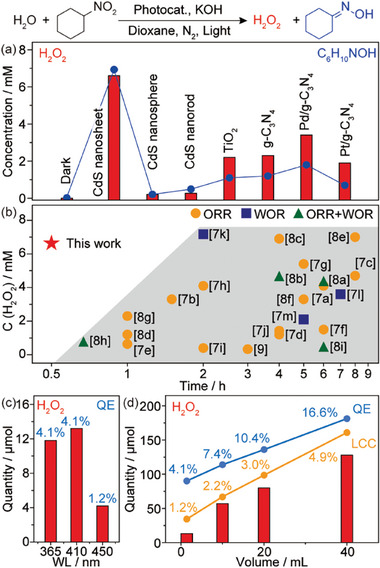
Photocatalytic performance. a) H_2_O_2_ evolution via WOR using different photocatalysts with nitrocyclohexane as acceptor (**A**) and dioxane as mediator (**M**). Reaction conditions: 10 mg photocatalyst in 2 mL 1 vol% water‐dioxane solution with 8 mm C_6_H_11_NO_2_ and 40 mM KOH under 410 nm irradiation (30 mW cm^−2^) and 1 bar N_2_ at RT for 0.5 h; b) Comparison of reported H_2_O_2_ evolution performances via ORR, WOR, and ORR+WOR, respectively; c,d) Effect of irradiation wavelength and volume of solution on H_2_O_2_ evolution using CdS nanosheets. Reaction conditions: 5 g L^−1^ photocatalyst in 1 vol% water‐dioxane solution with 8 mM C_6_H_11_NO_2_ and 40 mM KOH under 1 bar N_2_ at RT for 0.5 h. Light intensity: 30 mW cm^−2^. QE: quantum efficiency; LCC: light to chemical conversion efficiency.

Photocatalytic H_2_O_2_ evolution from WOR was performed in a 2 mL dioxane solution containing 1 vol% of water, 8 mm of nitrocyclohexane, and 40 mM of KOH at room temperature (RT) under 410 nm irradiation and deaerated conditions (**Figure** **2**a). The generated H_2_O_2_ and cyclohexanone oxime were quantified by UV–vis spectrometry and gas chromatography (GC), as described in the supplementary materials (Note S3 and Figures S7 and S8, Supporting Information). No reaction was observed in the absence of water nor under dark conditions in the presence of a photocatalyst, confirming that H_2_O_2_ solely originates from photocatalytic WOR (Table S1, Supporting Information). Remarkably, the concentration of H_2_O_2_ reached 6.6 mm within an irradiation time of 30 min when CdS nanosheets were used as photocatalysts (10 mg), according to both colorimetric and titration methods (Table S2, Supporting Information). This is accompanied by the production of an equal molar concentration of cyclohexanone oxime (C_6_H_10_NOH, ∼6.9 mM), suggesting a successful coupling of the 2e^‒^ water oxidation reaction with the 2e^‒^ reduction of nitrocyclohexane. The addition of KOH is also essential in achieving a high performance, indicating that H_2_O_2_ is generated via the oxidation of ionized water (OH^−^) into ^•^OH (1.99 V vs SHE).^[^ ^7l^ ^]^ Additionally, the cyclohexanone oxime is a value‐added product important for the polymer industry.^[^ ^17^ ^]^ A remarkable concentration of H_2_O_2_ (15.4 mM) can be achieved by using 1,8‐diazabicyclo[5.4.0]undec‐7‐ene (DBU) as the base (Entry 13; Table S1, Supporting Information). The high performance and the structure of the CdS nanosheet can be retained for 5 consecutive cycles and an additional cycle after a 24 h ageing of continuous irradiation, confirming the stability of the system for H_2_O_2_ synthesis (Figures S9 and S10, Supporting Information). However, the formation of cyclohexanone oxime is negligible (∼0.5 mM), owing to the preferential involvement of DBU in the hydrogenation half reaction.^[^ ^18^ ^]^ In comparison, CdS nanospheres and nanorods show poor catalytic performances with low conversion of nitrocyclohexane (∼1 mM, <12%, Figure S11, Supporting Information), possibly due to the mismatch of the band position. In comparison, a series of g‐C_3_N_4_ based photocatalysts were tested under similar reaction conditions, and in all cases show a high conversion of nitrocyclohexane and decreased selectivity to cyclohexanone oxime due to the formation of cyclohexanone (C_6_H_10_O, Figure S12, Supporting Information), resulting in a poor production of H_2_O_2_. Additionally, the strong oxidation power of TiO_2_ leads to the formation of various oxygen radicals,^[^ ^19^ ^]^ thus causing a low yield of H_2_O_2_. Nevertheless, the CdS nanosheet also displays an appropriate adsorption of H atoms that facilitates efficient donation of generated H atoms to **A**, which will be discussed hereafter.

The CdS nanosheets‐photocatalyzed WOR in a water‐dioxane‐nitrocyclohexane system outperforms most previously reported ORR, WOR, and ORR+WOR systems for the generation of H_2_O_2_, in terms of H_2_O_2_ concentration and efficiency (Figure 2b; Table S3, Supporting Information). High concentrations of H_2_O_2_ (>4 mm) are usually realized via the ORR path and requires a relatively long irradiation time (>4 h). In comparison, the WOR with an **A**‐**M** couple offers a solution for rapid evolution of concentrated H_2_O_2_ at RT. The CdS nanosheets show appreciable production of H_2_O_2_ under UV and visible light irradiation, with quantum efficiencies (QEs) of 4.1%, 4.1%, and 1.2% under 365, 410, and 450 nm irradiation in a 2 mL water‐dioxane‐nitrocyclohexane solution, respectively (Figure 2c). Furthermore, the amount of H_2_O_2_ produced via the **A**‐**M** couple‐promoted WOR increases monotonously upon expanding the reaction volume from 2 to 40 mL at a fixed irradiation time of 30 min (Figure 2d). A maximum QE of 16.6% is observed at 40 mL under 410 nm irradiation (Note S4, Supporting Information), demonstrating the enhanced utilization of photogenerated H atoms and ^•^OH radicals powered by the **A**‐**M** couple. This corresponds to a high light‐to‐chemical conversion (LCC) efficiency of 4.9% owing to the spontaneous cogeneration of cyclohexanone oxime (Note S5, Supporting Information).

### 2.3 Promotion Mechanisms

**Figure 3:**
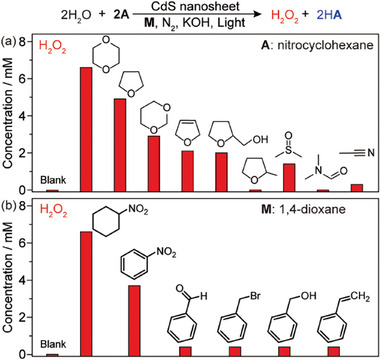
Effect of A and M. a) H_2_O_2_ evolution from WOR with nitrocyclohexane and different **M**. b) H_2_O_2_ evolution from WOR with different **A** and 1,4‐dioxane. Reaction conditions: 10 mg CdS nanosheets in 2 mL 1 vol% water‐**M** solution with 8 mm **A** and 40 mM KOH under 410 nm irradiation (30 mW cm^−2^) and 1 bar N_2_ at RT for 0.5 h.

The selection of the **A**‐**M** couple strongly influences the evolution of H_2_O_2_ (**Figure** **3**). Among a series of cyclic ethers, 1,4‐dioxane and tetrahydrofuran are identified as the most suitable mediators for coupling with nitrocyclohexane (Figure 3a), possibly due to their symmetric structures that may benefit the trapping of ^•^OH radicals.^[^ ^20^ ^]^ The reduced performances observed for tetrahydrofuran, 1,3‐dioxane, 2,5‐dihydrofuran, tetrahydrofurfuryl alcohol, and 2‐methyltetrahydrofuran are associated with the limited *α*‐C sites and possibly slower kinetics for the addition of ^•^OH radicals. In addition, unwanted oxidation products of these mediators are observed (Figure S13, Supporting Information), indicating an inefficient release of the trapped ^•^OH radicals from the mediator for the generation of H_2_O_2_. Other typical solvents including dimethyl sulfoxide (DMSO), *N,N*‐dimethyl formamide (DMF), and acetonitrile show poor performance for H_2_O_2_ evolution due to unsuccessful C─H bond activation. Alternatively, nitrocyclohexane and nitrobenzene are promising hydrogen acceptors to couple with 1,4‐dioxane (Figure 3b). Note that azoxybenzene is produced with high yield when nitrobenzene is employed as **A**, which is a valuable chemical for dye and electronic applications.^[^ ^21^ ^]^ The evolution of H_2_O_2_ is negligible when benzaldehyde and benzyl bromide are used as hydrogen acceptors, though an appreciable quantity of the corresponding reduction products (benzyl alcohol and bibenzyl) is observed (Figure S14, Supporting Information). This is likely caused by the rapid reverse reaction of the redox couples (benzyl alcohol/benzaldehyde) and the intermediates (Br^‒^/^•^OH radicals). The lack of a suitable cocatalyst for hydrogenation hinders the use of benzyl alcohol and styrene as **A**, thus resulting in a poor separation of H atoms from ^•^OH radicals for the generation of H_2_O_2_.

**Figure 4:**
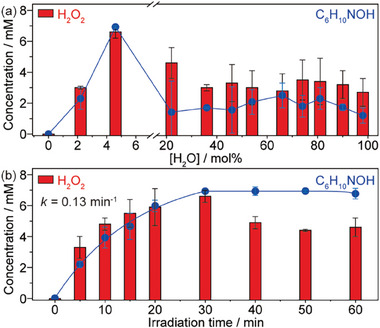
Promotional mechanisms. a) Effect of water concentration on the photocatalytic WOR. b) Evolution of H_2_O_2_ and cyclohexanone oxime from a 1 vol% water‐dioxane solution. Reaction conditions: 10 mg CdS nanosheets in 2 mL water‐dioxane with 8 mm C_6_H_11_NO_2_ and 40 mM KOH under 410 nm irradiation (30 mW cm^−2^) in 1 bar N_2_ at RT.

The effect of water concentration and the reaction kinetics of H_2_O_2_ evolution are further explored using nitrocyclohexane and dioxane as **A** and **M**, respectively (**Figure** **4**). Neither H_2_O_2_ nor C_6_H_10_NOH was observed in the absence of water, confirming that both H atoms and ^•^OH radicals solely originate from water (Figure 4a). The yield of H_2_O_2_ and C_6_H_10_NOH reached an optimum at a water concentration of ∼5 mol% (1 vol%) but decreased upon further increasing the water content. A relatively low concentration of C_6_H_10_NOH implies that the hydrogenation half reaction limits the overall reaction. This also indicates that isolated water molecules in a diluted dioxane solvent are more prone to dissociate, possibly due to the weakening of intermolecular hydrogen bonds.^[^ ^7k^ ^]^ The evolution of both H_2_O_2_ and C_6_H_10_NOH follows *pseudo* first order kinetics within the first 30 min of irradiation with a similar rate constant (*k* = 0.13 min^−1^, Figure 4b; Figure S15, Supporting Information). While the concentration of C_6_H_10_NOH remains constant at prolonged irradiation times, a slight decomposition of H_2_O_2_ is observed which eventually stabilized at ∼4.6 mm after 60 min.

**Figure 5:**
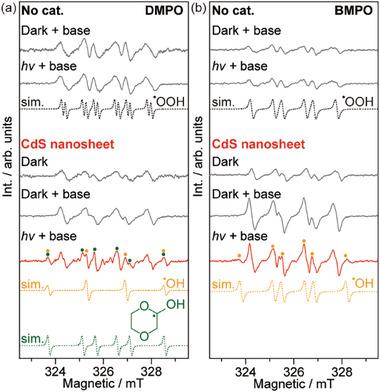
Promotional mechanisms. a,b) EPR spectra of the photocatalyst‐dioxane suspension probed using DMPO and BMPO spin trapping agents. Reaction conditions: 10 mg catalyst in 0.5 mL of 1 vol% 4 m KOH‐dioxane with 20 mM spin trap under deaerated conditions, irradiated by a 410 nm LED at RT for 1 min.

Electron paramagnetic resonance (EPR) was employed to identify the formation of ^•^OH radical species using 5,5‐dimethyl‐1‐pyrroline *N*‐oxide (DMPO) and 5‐tert‐butoxycarbonyl‐5‐methyl‐1‐pyrroline‐*N*‐oxide (BMPO) as the spin trap in a 1 vol% 4 M KOH‐dioxane solution (**Figure** **5**a,b). Silent spectra were observed under both dark and irradiation conditions in the absence of a photocatalyst and KOH (Figure S16, Supporting Information). Interestingly, the characteristic DMPO−^•^OOH adduct is observed in the absence of CdS nanosheets in basic media regardless of dark or light conditions (*g*_iso_ = 2.004, *α*_iso_(^14^N) = 37.66 MHz, and *α*_iso_(^1^H_β_) = 24.26 and *α*_iso_(^1^H_γ_) = 4.27 MHz).^[^ ^20b^ ^]^ This DMPO−^•^OOH adduct originates from the peroxides that are generated from 1,4‐dioxane reacting with residual O_2_.^[^ ^22^ ^]^ It is worth noting that the EPR spectra remains unchanged with the addition of CdS nanosheets under dark conditions, indicating that the ^•^OOH radical is unrelated to the primary catalytic reaction of interest. Upon irradiation, there is evidence for the evolution of both DMPO‐^•^OH (*g*_iso_ = 2.004, *α*_iso_(^14^N) = 44.96 MHz and *α*_iso_(^1^H_β_) = 44.96 MHz) and a small amount of DMPO‐^•^ketyl adducts (*g*_iso_ = 2.004, *α*_iso_(^14^N) = 39.90 MHz and *α*_iso_(^1^H_β_) = 55.16 MHz).^[^ ^23^ ^]^ The detection of ketyl radical is a clear evidence of the dioxane‐captured ^•^OH radical, which originates from photocatalytic dissociation of water prior to the formation of H_2_O_2_. This is evidenced by the formation of 1,4‐dioxane‐2‐ol under neutral and acidic conditions upon irradiation (Figure S17a, Supporting Information), which is the stabilized form of the dioxane‐ketyl radical. Additionally, a previous work also shows that the DMPO−^•^OOH is unlikely to be the origin of DMPO‐^•^OH.^[^ ^24^ ^]^ Irradiation of the CdS‐dioxane suspension in acidic conditions with the presence of H_2_O, D_2_O, and H_2_^18^O results in the formation of labeled 1,4‐dioxane‐2‐ol molecules (Figure S17b, Supporting Information), confirming that the origin of generated ^•^OH radicals is from water oxidation. In comparison, no ketyl radicals are observed when CdS nanorods and nanospheres are used under irradiation (Figure S18, Supporting Information). The evolution of radicals with shorter lifetimes has been further verified by using BMPO as an alternative spin trap (Figure 5b).^[^ ^25^ ^]^ Only ^•^OOH radicals are observed in the absence of the CdS or under dark conditions, agreeing well with the DMPO data. Similarly, evidence of both BMPO‐^•^OH (*g*_iso_ = 2.005, *a*_iso_(^14^N) = 37.38 MHz, *a*_iso_(^1^H_β_) = 47.92 MHz, *a*_iso_(^1^H_γ_) = 1.74 MHz) and BMPO−^•^OOH (*g*_iso_ = 2.005, *a*_iso_(^14^N) = 36.50 MHz, *a*_iso_(^1^H_β_) = 26.42 MHz) adducts are observed upon irradiation of the CdS nanosheets.^[^ ^25^ ^]^ The presence of hydroxyl radicals indicates the release of captured ^•^OH from the **M** ^•^OH prior to the formation of H_2_O_2_, thus rationalizing the choice of 1,4‐dioxane as a suitable mediator for WOR. In contrast, negligible ^•^OH radicals are observed for the CdS nanorods and nanospheres (Figure S18, Supporting Information). Additionally, the evolution of ^•^OH radicals and H species is also confirmed by dosing 2,2,6,6‐tetramethylpiperidinyloxy (TEMPO) in the reaction solution (Figure S19, Supporting Information).

**Figure 6:**
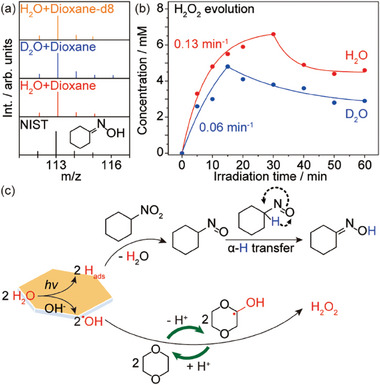
Promotional mechanisms. a) Photocatalytic reduction of nitrocyclohexane with the presence of H_2_O, D_2_O, and 1,4‐dioxane‐d_8_. b) Kinetics of hydrogen peroxide evolution using H_2_O and D_2_O, respectively. Reaction conditions: 10 mg CdS nanosheets in 2 mL 1 vol% water‐dioxane solution with 8 mM nitrocyclohexane and 40 mM KOH under 410 nm irradiation (30 mW cm^−2^) and 1 bar N_2_ at RT. c) Reaction pathways for H_2_O_2_ evolution from photocatalytic WOR with an **A**‐**M** couple.

The destination of the photogenerated H atoms is verified by mass spectrometry (MS) analysis of the photogenerated cyclohexanone oxime via isotopic labeling experiments using H_2_O, D_2_O, and 1,4‐dioxane‐d_8_ (**Figure** **6**a). Interestingly, the MS patterns are almost identical and match well with the standard spectrum of cyclohexanone oxime from the National Institute of Standards and Technology (NIST),^[^ ^26^ ^]^ with only a small trace observed at M/e = 116 for the D_2_O case. Since the reaction involves a hydrogen atom transfer step and the presence of surface trapped H atoms is observed according to the appearance of CdS nanosheets (Figure S20, Supporting Information),^[^ ^21a^ ^]^ it follows that the H atom abstracted from water is involved in the reduction of nitrocyclohexane to the nitrosocyclohexane intermediate with water as the byproduct. We have further analyzed the isotopic effect in the evolution kinetics of hydrogen peroxide using deuterated water (Figure 6b). A k_H_/k_D_ of 2.17 confirms a primary kinetic isotope effect, suggesting the rate determining step is the breaking of the H─O bond in water.

The mechanism of the **A**‐**M** couple promoted photocatalytic H_2_O_2_ evolution via WOR using CdS nanosheets is thus proposed in Figure 6c. Upon irradiation, the CdS nanosheets offer suitable band positions for the oxidation of OH^‒^ (ionized in basic media) and the reduction of H^+^ into ^•^OH radicals and surface adsorbed H atoms (H_ads_), respectively. The nitrocyclohexane serves as a hydrogen acceptor, which consumes the H_ads_ rapidly via the formation of the nitrosocyclohexane intermediate and release of H_2_O simultaneously. The unstable nitrosocyclohexane undergoes an *α*‐H transfer tautomerization to yield cyclohexanone oxime.^[^ ^27^ ^]^ Meanwhile, the 1,4‐dioxane serves as an ^•^OH mediator, which extends the lifetime of the ^•^OH radical via formation of ketyl radicals through a dehydrogenative step in basic conditions under deaerated conditions. The ketyl radical reacts with another ketyl radical or an ^•^OH radical to generate H_2_O_2_ thus regenerating dioxane together with the released proton.

## 3 Conclusion

In summary, we demonstrate that efficient photocatalytic H_2_O_2_ evolution from WOR can be realized under basic conditions by employing an acceptor‐mediator (**A**‐**M**) couple to consume the hydrogen atom and prolong the lifetime of ^•^OH radicals. Specifically, the nitrocyclohexane‐dioxane‐CdS nanosheet system enables a rapid kinetic production of H_2_O_2_ (0.13 min^−1^) to a high concentration (6.6 mm), featuring a high QE (16.6%) and light to chemical conversion efficiency (LCC, 4.9%) under 410 nm irradiation. The dioxane mediator stabilizes ^•^OH radicals via the formation of ketyl radicals prior to the generation of H_2_O_2_, and the nitrocyclohexane rapidly consumes the H atoms to yield cyclohexanone oxime, which is a value‐added precursor of Nylon 6. This reaction proceeds readily at room temperature and with good recyclability, thus rendering it a potential option for on‐site H_2_O_2_ production. The H_2_O_2_‐dioxane solution could be employed for catalytic epoxidation, oxidation, and hydroxylation reactions after tweaking the reaction parameters, though the development of a photocatalytic system that can generate aqueous H_2_O_2_ solution with very minor impurities is preferred.

## Conflict of Interest

The authors declare no conflict of interest.

## Supporting information



Supporting Information

## Data Availability

The data that support the findings of this study are available in the supplementary material of this article.
