# Supplementary material for: Photocatalytic Partial Water Oxidation Promoted by a Hydrogen Acceptor‐Hydroxyl Mediator Couple
Source: Adv Sci (Weinh). 2024 Dec 20;12(6):2410680. doi: 10.1002/advs.202410680 (PMC11809376; doi:10.1002/advs.202410680)
Supplement: Supplementary file 1 — Supporting Information [file ADVS-12-2410680-s001.docx]

Supporting Information
©Wiley-VCH 2021
69451 Weinheim, Germany

Photocatalytic Partial Water Oxidation Promoted by a Hydrogen Acceptor-Hydroxyl Mediator Couple

Yuanqiang Mai,^[a],#^ Dongsheng Zhang,^[a],[b],#^ Kristina Maliutina,^[c],#^ Xueyang Leng,^[a],#^ Nengjun Cai,^[a]^ Jialu Li,^[a]^ Chao Wang,^[a]^ Yu Huang,^[b],[d]^ Kai Zhang,^[b],[d]^ Wujun Zhang,^[e]^ Yongwang Li,^[b],[f]^ Flemming Besenbacher,^[g]^ Hans Niemantsverdriet,^[b],[h]^ Wenting Liang,^[d]^ Yanbin Shen,^[e]^ Tingbin Lim,^[i]^ Emma Richards, ^[c]^* and Ren Su ^[a],[b]^*

**Abstract:** Hydrogen peroxide (H_2_O_2_) is an important chemical in synthetic chemistry with huge demands. Photocatalytic synthesis of H_2_O_2_ *via* oxygen reduction and water oxidation reactions (ORR and WOR) is considered as a promising and desirable solution for on-site applications. However, the efficiency of such a process is low due to the poor solubility of molecular oxygen and the rapid reverse reaction of hydroxyl radicals (^•^OH) with hydrogen atoms (H). Here, we proposed a strategy to boost the H_2_O_2_ evolution *via* oxidation of water by employing a H acceptor (**A**, nitrocyclohexane), an ^•^OH mediator (**M**, dioxane) and a photocatalyst (CdS nanosheets). While ^•^OH radicals are stabilized by dioxane to produce ketyl radicals prior to the formation of H_2_O_2_, H atoms are effectively utilized in the generation of cyclohexanone oxime, an important intermediate in the production of Nylon 6. The system displays a rapid kinetic accumulation of H_2_O_2_ (0.13 min^-1^) to a high concentration (6.6 mM). At optimum reaction conditions, a high quantum efficiency (16.6%) and light-to-chemical conversion efficiency (4.9%) can be achieved under 410 nm irradiation.

DOI: 10.1002/anie.2021XXXXX

Table of Contents

1. Supplementary Notes 3

2. Supplementary Tables 5

3. Supplementary Figures 7

4. References 14

Supplementary Notes

Note S1. Synthetic protocol of photocatalysts.

All reagents and solvents were used without further purification unless otherwise stated.

**Synthesis of CdS nanosphere:** In a typical synthesis, 2.4 mmol of Cd(OAc)_2_·2H_2_O and 60 mmol of CH_4_N_2_S were dissolved in 60 mL of deionized (DI) water to form a homogeneous solution following sonication for 5 min and stirring for 30 min at room temperature (RT). Then, the solution was transferred to an 80 mL Teflon-lined stainless-steel autoclave and maintained at 140 ^o^C for 5 h. The orange precipitate was collected by centrifugation, washed by DI water and ethanol three times, and eventually freeze-dried.

**Synthesis of CdS nanorod:** 15 mmol of Cd(NO_3_)_2_·2H_2_O and 45 mmol of CH_4_N_2_S were dissolved in 60 mL of ethylenediamine to form a homogeneous solution following sonication for 5 min and stirring for 30 min at RT. Then, the solution was transferred to an 80 mL Teflon-lined stainless-steel autoclave and maintained at 160 ^o^C for 48 h. The dark yellow solid was collected by centrifugation, washed by DI water and ethanol three times, and dried at 60 ^o^C in vacuum overnight.

**Synthesis of CdS nanosheet:** 2 mmol of Cd(OAc)_2_·2H_2_O and 6 mmol of CH_4_N_2_S were dispersed in 60 mL of ethylenediamine, followed by sonication for 30 min and stirring for 30 min at RT. The mixture was transferred to an 80 mL Teflon-lined stainless-steel autoclave and heated at 100 ^o^C for 8 h. The bright yellow solid was collected by centrifugation, washed by DI water and ethanol three times, and dried at 60 ^o^C in vacuum overnight.

Note S2. Characterizations

X-ray diffraction (XRD) patterns were recorded on a Bruker D8 Advance diffractometer in the scan range of 5 − 80° with a step size of 0.02° and an integration time of 0.2 s using a Cu- Kα radiation source (40 kV, 40 mA). Transmission electron microscopy (TEM, Titan Themis Cubed G2 300) was employed to analyze the morphology of CdS photocatalysts. The powder samples were dispersed in ethanol and dropped onto Cu grids for analysis. The chemical compositions and oxidation state of elements on the surface region of the CdS photocatalysts were analyzed by X-ray photoelectron spectroscopy (XPS) using an Al Kα X-ray source (Thermo Fisher Scicentific, USA). Survey scans were measured from 1200 to -10 eV using a pass energy of 160 eV with a step size of 1 eV and a dwell time of 0.1 s, whereas the region-of-interest spectra (C1s, O1s, Cd3d and S2p) were collected using a pass energy of 40 eV with a step size of 0.1 eV and a dwell time of 0.5 s. The adventitious carbon was used for calibration (C1s = 284.6 eV). Diffuse reflectance spectra (DRS) of the photocatalysts were measured using an UV-vis spectrophotometer (UV 2600, Shimadzu) equipped with an integrating sphere. The DRS data were recorded in the range of 200-800 nm employing BaSO_4_ as the reference. The bandgap of the photocatalysts was calculated using the Kubelka-Munk theory.^[28]^ Mott-Schottky plots of the photocatalysts were recorded on a DH7000C electrochemical workstation (DongHua Testing Technology, China). A Pt wire and a saturated Ag|AgCl were employed as the counter electrode (CE) and reference electrode (RE), respectively. The working electrode (WE) was assembled by coating the photocatalyst powders on an indium-tin oxide glass (ITO). A 10 mg of photocatalyst powders was homogeneous dispersed in 950 μL ethanol that contains 50 μL 5% Nafion solution. Then a 30 μL of the suspension was dropped on the ITO glass and dried at 60 ^o^C under vacuum for 4 h. The electrolyte was a 0.5 M Na_2_SO_4_ aqueous solution. The scanning voltage range was ±1 V + open-circuit voltage (OCV), which was measured prior to analysis. The amplitude bias was 5 mV with a frequency of 1000 Hz. The radical species generated in the photocatalytic reaction were analyzed by electron paramagnetic resonance (EPR) spectroscopy using a JES-X320 spectrometer in the range of 321-331 mT at 298 K. The amplitude is 400 and the mod width is 0.1 mT in all measurements. 5,5-Dimethyl-1-pyrroline N-oxide (DMPO) or 5-tert-Butoxycarbonyl-5-methyl-1-pyrroline-N-oxide (BMPO) were used as spin trapping agents, forming spin-trapped adducts with short-lived organic radical species. Simulations of EPR spectra were performed using the garlic function in the Easyspin toolbox for Matlab.^[29]^ Samples for EPR spectroscopy measurements were prepared as follows: 10 mg catalyst was dispersed in 400 μL 1,4-dioxane with stirring overnight. Immediately prior to data acquisition, an additional 100 μL 1,4-dioxane that contains 100 mM spin trap and 5 μL 4 M KOH were dosed into the suspension. The EPR spectra were recorded under “dark” (no irradiation) and “light” conditions (after irradiation at 410 nm LED for 1 min).

Note S3. Photocatalytic performance.

**The 2 mL system:** 16 μmol nitrocyclohexane (C_6_H_11_NO_2_), 20 μl 4 M KOH solution ,10 mg catalyst powders, and 2 mL dioxane were added into a 4 mL glass reactor. The reaction suspension was purged for two minutes by nitrogen gas. A 410 nm annular LED was used as the light source (30 mW·cm^−2^). The suspension was centrifuged at desired reaction times, and the liquid was extracted for analysis.

The quantity of photogenerated H_2_O_2_ was quantified by a colorimetric titration method employing a Cu(II) based chromogenic agent. ^[30]^ The chromogenic agent was prepared by mixing a 2,9-dimethyl-1,10-phenanthroline (DMP)-ethanol solution (1 g·L^−1^) with an aqueous CuSO_4_ solution (0.01 M) in a 1:1 volume ratio. For analysis, 50 μl of aliquots from the reaction were added into 3 mL of the freshly prepared chromogenic agent solution. The absorption spectra of the well-mixed solution were measured by a UV-vis spectrophotometer (UV 2600, Shimadzu) to determine the concentration of H_2_O_2_. The molar absorption coefficient (α) of the reduced chromogenic agent (Cu(I)-DMP complex) by H_2_O_2_ at 454 nm is determined to be 7 mM^-1^·cm^−1^ according to the calibration curve using a series of standard H_2_O_2_ solution with known concentrations (Fig. S6). Note that one part of H_2_O_2_ can reduce two parts of Cu(Ⅱ)-DMP into Cu(Ⅰ)-DMP. The photogenerated cyclohexanone oxime (C_6_H_10_NOH) can also reduce the chromogenic agent slowly, causing change of absorbance at 454 nm. The exact stoichiometry of C_6_H_10_NOH induced reduction of Cu(II)-DMP is still unclear, but is more than 2 according to a previous work. ^[31]^ Therefore, we have prepared a series of standard C_6_H_10_NOH solution to determine the influence of C_6_H_10_NOH on the absorption of the chromogenic agent. A very small slope is observed at 454 nm (8.9×10^−2^ mM^−1^·cm^−1^ ), which is ~two orders of lower than that of H_2_O_2_, thus the impact of C_6_H_10_NOH in the reaction solution can be neglected. Additionally, the concentration of H_2_O_2_ has been also determined by a standard titration method using potassium permanganate (Table S3).^[32]^ The organic compounds were analyzed by gas chromatography (GC, Agilent 8860) and gas chromatography-mass spectrometry (GC-MS, Agilent 8860 GC coupled with a 5977B mass selective detector). The selectivity (Sel.) and yield were determined by GC.

**The larger volume systems:** The desired volumes of dioxane (10, 20, 40 mL and 100 mL) that contain 8 mM C_6_H_11_NO_2_, 1 vol% 4 M KOH, and 5 g·L^−1^ photocatalyst were added into a glass reactor. The reaction suspension was purged for five minutes by nitrogen gas. A 410 nm annular LED with a maximum output power of 250 mW was used. The suspension was centrifuged at desired reaction times, and the liquid was extracted for analysis.

Note S4. Estimation of quantum efficiency (QE)

The QE for photocatalytic generation of H_2_O_2_ is estimated according to Eq. S1:

$QE=\frac{n_{product}\times n_{e,i}\times N_{A}}{N_{1h}\times t_{R}}\times100 \%$ Eq. S1

where n_product_ is the amount of produced H_2_O_2_ (mol) determined from titration, n_e,i_ is the number of transferred electrons to generate one H_2_O_2_ molecule from water oxidation (2). N_A_ is the Avogadro's constant (6.02×10^23^ mol^−1^), t_R_ is the irradiation time.

The number of incident photons per hour (N_1h_) is calculated according to Eq. S2:

$N_{1h}=\frac{E\times\lambda\times t}{h\times c}=\frac{W_{lamp}\times S_{R}\times\lambda\times t}{(6.626\times{10}^{-34} J\cdot s)\times(3\times{10}^{8} m\cdot s^{1})}$ Eq. S2

where W_lamp_ is the light intensity of LED lamp (30 mW·cm^−2^), S_R_ is the effective irradiation area (3.5 cm^2^ for 2 mL). For larger volume systems (>10 mL), the S_R_ is large enough to absorb all incident light, thus the maximum output power of 250 mW was used. λ is the wavelength of the light (410 nm), t is irradiation time (3600 s), h is the Planck constant (6.626 × 10^-34^ J·s) and c is the speed of light (3.0 × 10^8^ m·s^−1^). The estimated N_1h_ of the 2 mL system and larger volume systems are 7.8×10^20^ and 1.9×10^21^, respectively.

Note S5. Calculation of light to chemical conversion efficiency (LCC)

Photocatalytic H_2_O oxidation coupled with nitrocyclohexane reduction for the synthesis of H_2_O_2_ and cyclohexanone oxime is described as Eq. S3:


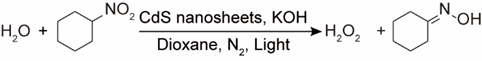
 Eq. S3

The LCC efficiency (η) of this reaction is calculated according to Eq. S4:

$\eta\left( \% \right)=\frac{\Delta_{r}H_{m}\times n}{E\times t_{R}}=\frac{\Delta_{r}H_{m}\times n}{W_{lamp}\times S_{R}\times t_{R}}\times100\%$ Eq. S4

where n is the number of generated H_2_O_2_/cyclohexanone oxime (in mol). The molar enthalpy of this reaction (∆_r_H_m_) is estimated according to Eq. S5:

$\Delta_{r}H_{m}=\Delta_{f}H_{m}\left( C_{6}H_{10}NOH,l \right)+\Delta_{f}H_{m}\left( H_{2}O_{2},l \right)-\Delta_{f}H_{m}\left( C_{6}H_{11}NO_{2},l \right)-\Delta_{f}H_{m}\left( H_{2}O,l \right)$ Eq. S5

where ∆_f_H_m_ (C_6_H_10_NOH, l), ∆_f_H_m_ (H_2_O_2_, l), ∆_f_H_m_ (C_6_H_11_NO_2_, l), and ∆_f_H_m_ (H_2_O, l) are -141.5 kJ·mol^-1^, -187.78 kJ·mol^-1^, -214.04 kJ·mol^-1^, and -285.83 kJ·mol^-1^ under standard pressure (101.325 kPa) and temperature (298.15 K), respectively. ^[33]^

Supplementary Tables

**Table S1.** Control experiments for photocatalytic generation of H_2_O_2_.^[a]^


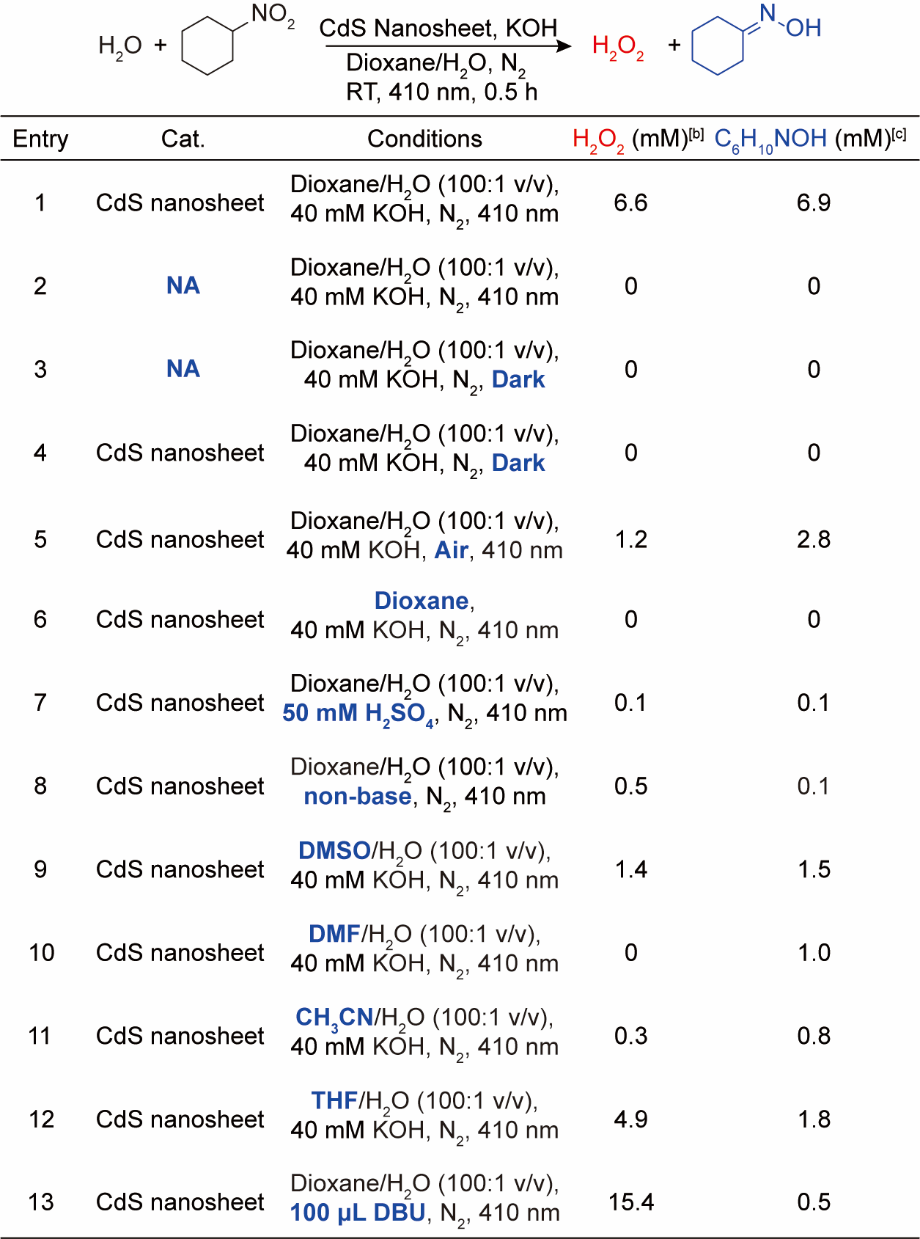


[a] Reaction conditions: C_6_H_11_NO_2_ (16 μmol), 10 mg photocatalyst in 1,4-dioxane (2 mL) at RT for 0.5 h under N_2_; [b] Determined by colorimetric titration method; [c] GC yield

**Table S2.** Concentrations of H_2_O_2_ determined by colorimetric analysis and titration.

| Sample ID | No. | [H_2_O_2_] / mM  by colorimetry | [H_2_O_2_] / mM  by titration |
| --- | --- | --- | --- |
| 100 mM  H_2_O_2_ solution | Test 1 | 97.2 | 108.0 |
|  | Test 2 | 99.8 | 109.0 |
| Reaction  solution | Test 1 | 6.6 | 8.0 |
|  | Test 2 | 7.1 | 9.0 |

**Table S3.** Comparison of reported H_2_O_2_ production via ORR and WOR.^[34]^


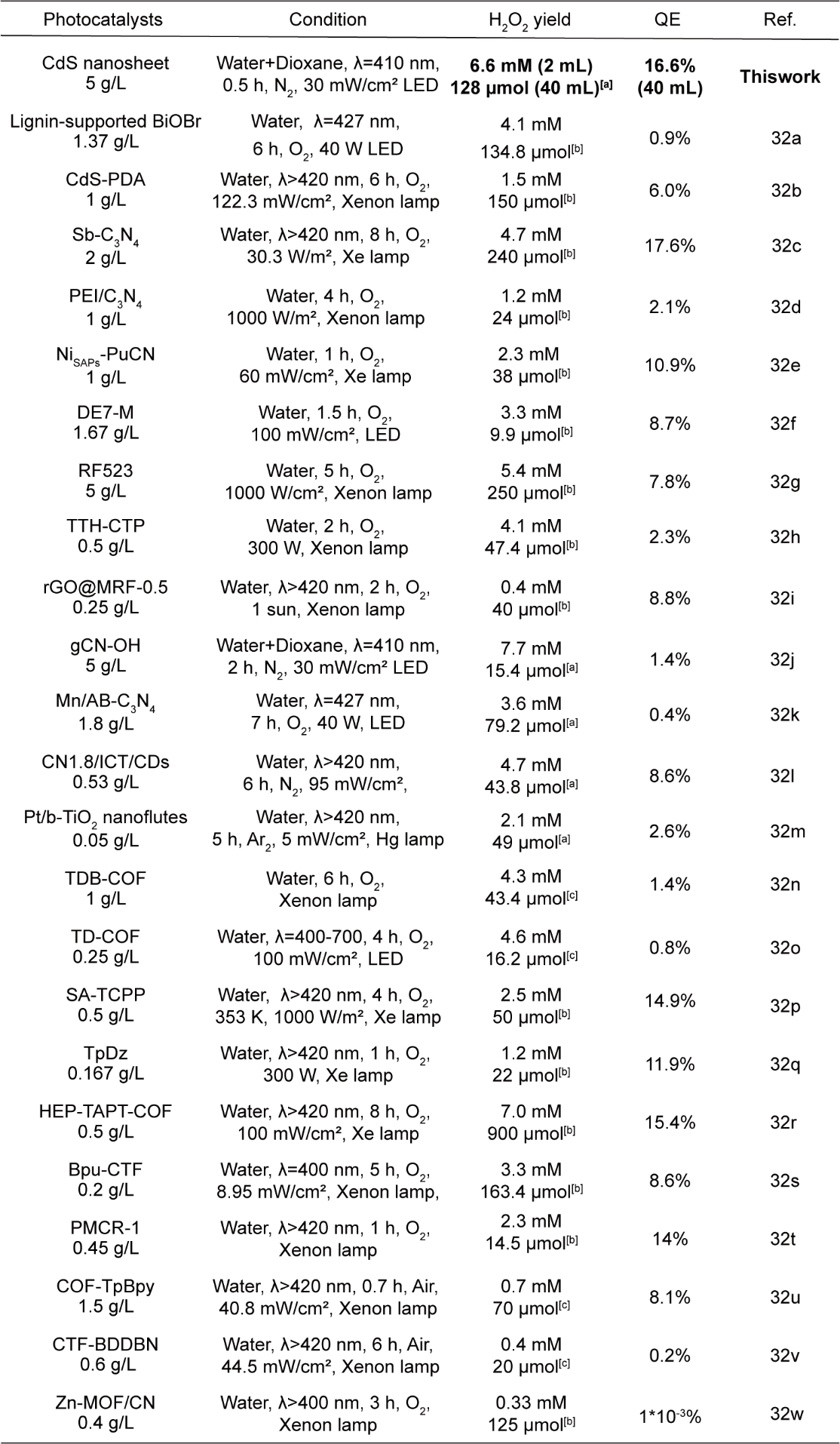


[a] WOR; [b] ORR; [c] ORR+WOR.

Supplementary Figures

The morphology of CdS nanosheets, nanorods, and nanospheres by scanning electron microscopic imaging is shown in Fig. S1 (SEM, Thermo Scientific Scios).


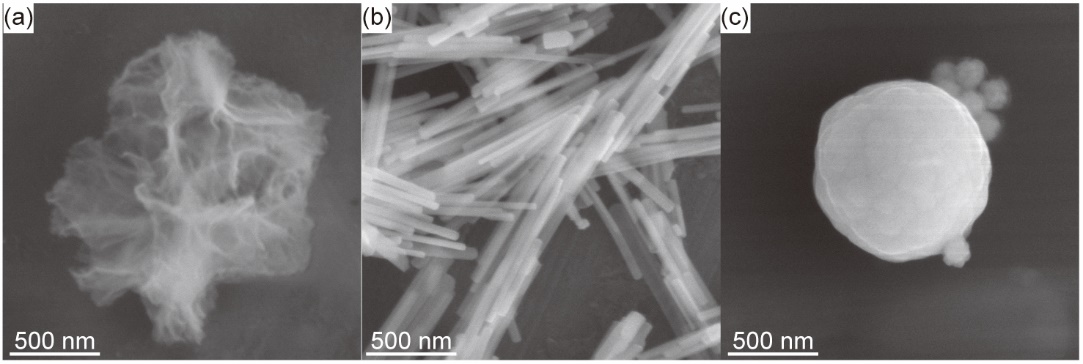


**Figure S1.** (a)-(c) SEM images of CdS nanosheets, nanorods, and nanospheres.

The TEM, XRD, and XPS characterizations of CdS nanorods and nanospheres are shown in Figs. S2 and S3.


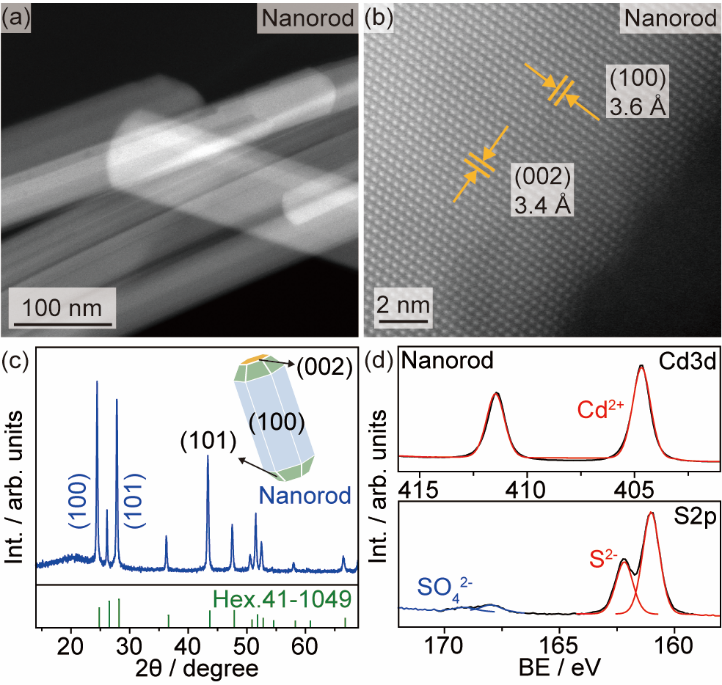


**Figure S2.** Characterization of the CdS nanorods by (a) and (b) TEM, (c) XRD, and (d) XPS.


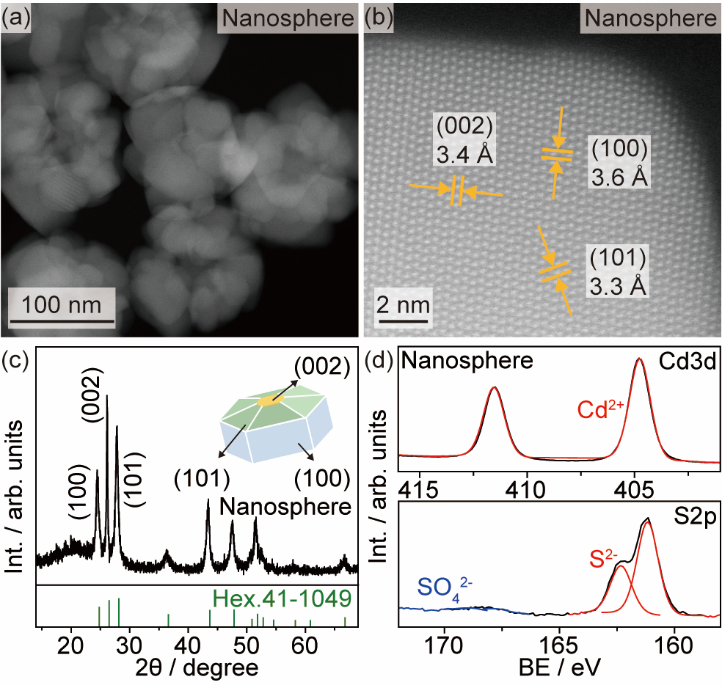


**Figure S3.** Characterization of the CdS nanospheres by (a) and (b) TEM, (c) XRD, and (d) XPS.

The XPS survey, O1s and C1s spectra of all CdS photocatalysts are shown in Fig. S4.


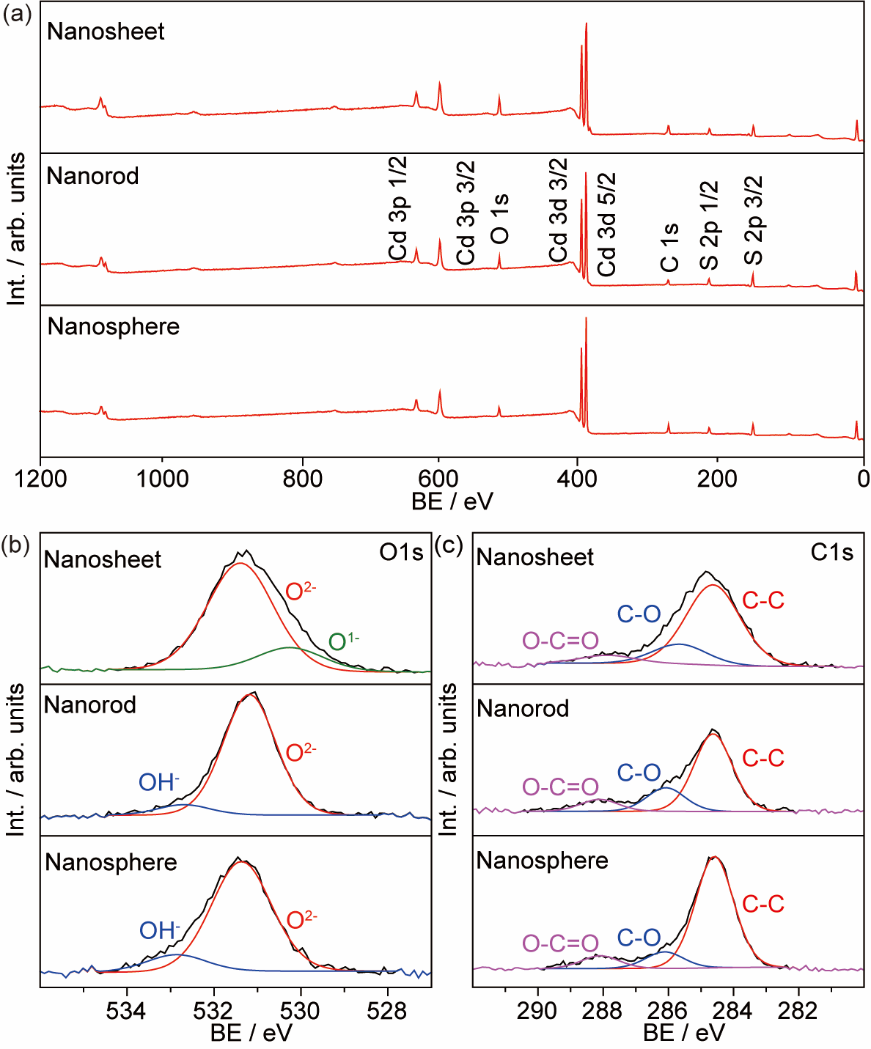


**Figure S4.** XPS of all CdS photocatalysts. (a) Survey spectra; (b) and (c) O1s and C1s spectra.


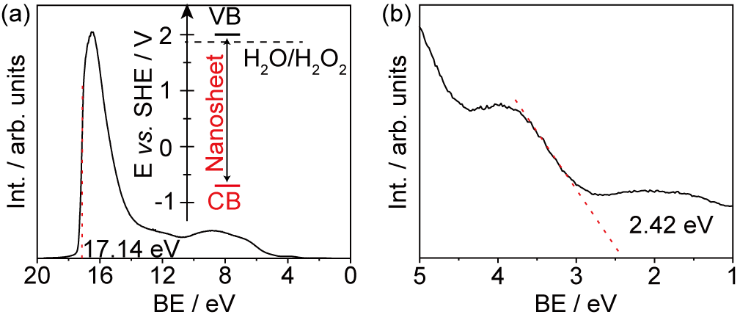


**Figure S5.** UPS of the CdS nanosheet and derived VB.

The N_2_ adsorption isotherms of all CdS photocatalysts were measured at 77 K using a Micromeritics ASAP 2460 system, as shown in Fig. S5. The specific surface areas were calculated by applying the Brunauer-Emmett-Teller (BET) equation to the isotherms in the relative pressure range of 0.05 to 0.3. The specific surface areas of CdS nanospheres, nanorods and nanosheets were 8.1, 21.7 and 94.3 m^2^·g^-1^, respectively.


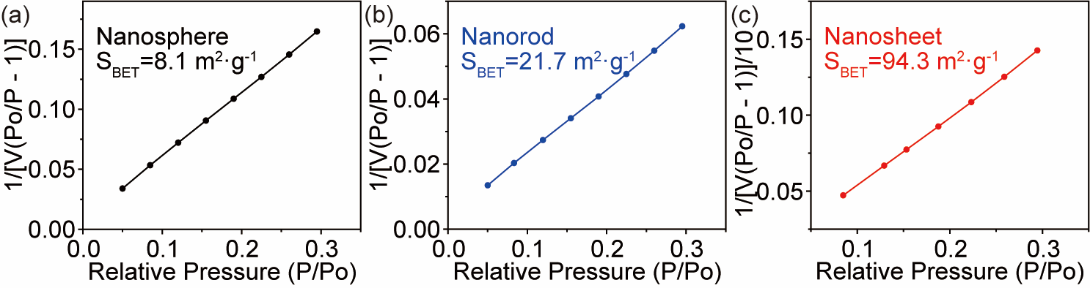


**Figure S6.** BET plots of the N_2_ adsorption isotherms for (a) nanosphere, (b) nanorod and (c) nanosheet CdS photocatalysts.

The UV-vis spectra for the determination of molar absorption coefficient (α) of the reduced chromogenic agent (Cu(I)-DMP complex) by H_2_O_2_ and C_6_H_10_NOH are shown in Figs. S7.


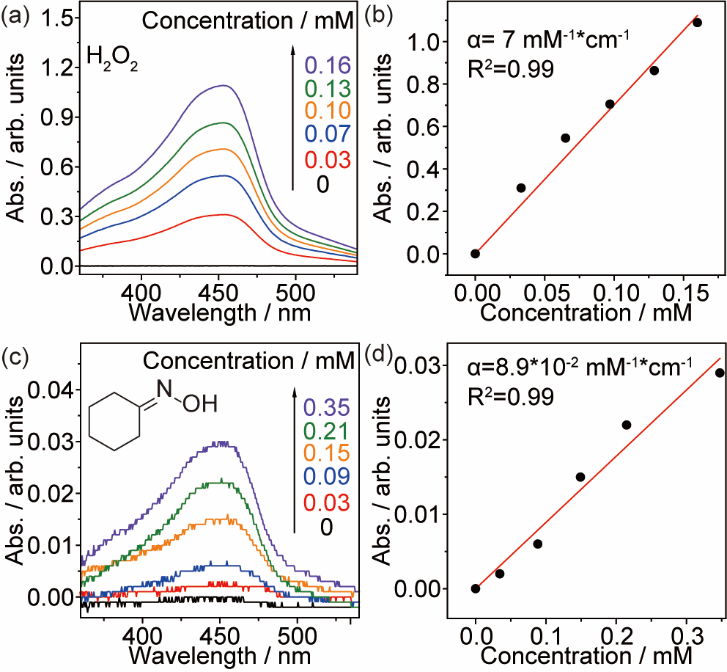


**Figure S7.** Colorimetric titrations of H_2_O_2_ and C_6_H_10_NOH. (a) and (b) UV-vis spectra of H_2_O_2_ at given concentrations within the chromogenic agent solution and the derived absorption coefficient of Cu(I)-DMP complex at 454 nm. (c) and (d) The effect of C_6_H_10_NOH on the chromogenic agent solution.

The UV-vis spectra for the determination of molar absorption coefficient (α) of the reduced chromogenic agent (Cu(I)-DMP complex) by H_2_O_2_ with the present of DBU are shown in Figs. S8.


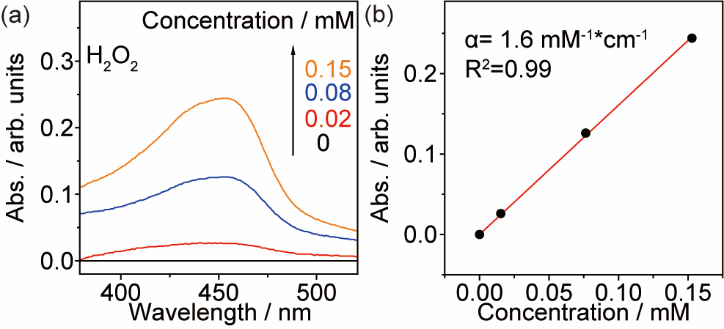


**Figure S8.** Colorimetric titrations of H_2_O_2_ in the present of DBU (a) and (b) UV-vis spectra of H_2_O_2_ at given concentrations within the chromogenic agent solution with DBU and the derived absorption coefficient of Cu(I)-DMP complex at 454 nm.


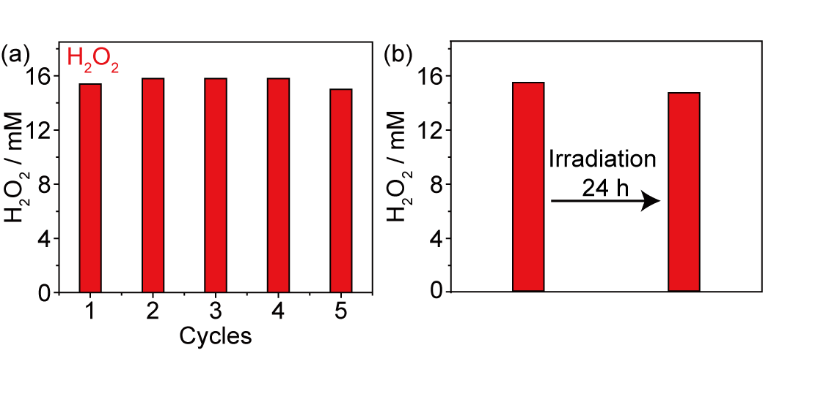


**Figure S9.** Stability of CdS in (a) photocatalytic H_2_O_2_ production for five consecutive cycles; and (b) catalytic performance of the spent CdS nanosheet after a 24-h irradiation in the reaction solution. Reaction conditions: 10 mg photocatalyst in 2 ml 1 vol% water-dioxane solution with 8 mM C_6_H_11_NO_2_ and 100 μL DBU under 410 nm irradiation (30 mW·cm^−2^) at RT. Each cycle is irradiated for 0.5 h during the stability test.


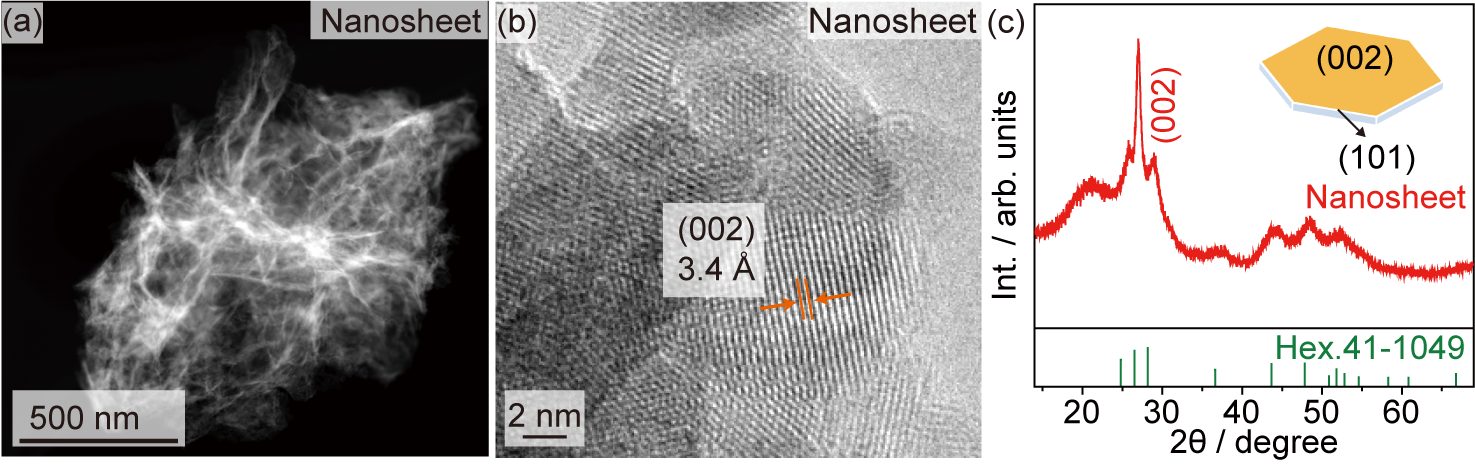


**Figure S10.** Characterizations of the spent CdS nanosheet by (a) and (b) TEM, and (c) XRD (cf Figure 1a,b,c of the fresh catalyst).


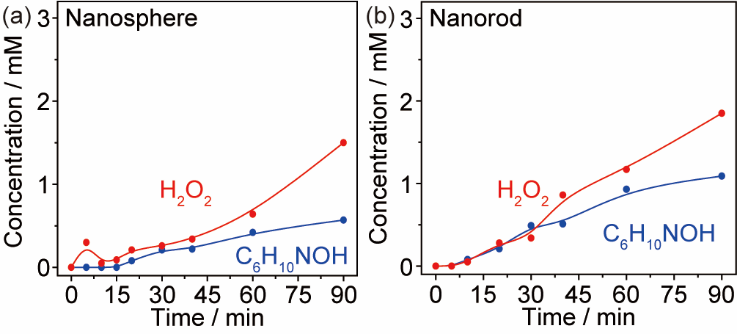


**Figure S11.** The evolution of H_2_O_2_ and C_6_H_10_NOH using (a) CdS nanosphere, and (b) CdS nanorod photocatalysts. Reaction conditions: 10 mg photocatalyst in 2 ml 1 vol% water-dioxane solution with 8 mM C_6_H_11_NO_2_ and 40 mM KOH under 410 nm irradiation (30 mW cm^-2^) and 1 bar N_2_ at RT.


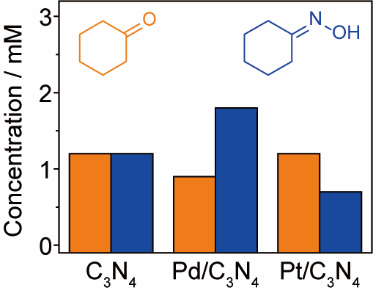


**Figure S12.** The catalytic performance of g-C_3_N_4_ based photocatalysts. Reaction conditions: 10 mg photocatalyst in 2 ml 1 vol% water-dioxane solution with 8 mM C_6_H_11_NO_2_ and 40 mM KOH under 410 nm irradiation (30 mW·cm^−2^) at RT for 0.5 h.


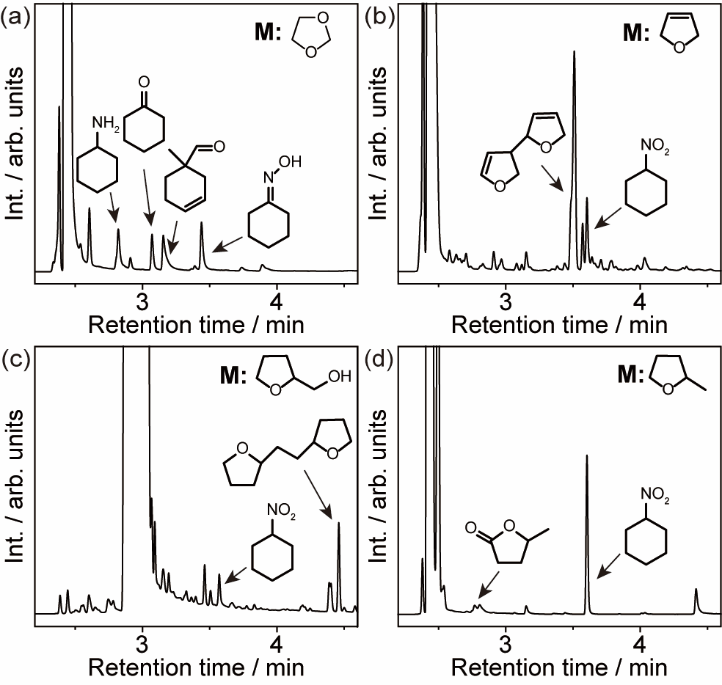


**Figure S13.** GC spectra of photocatalytic H_2_O_2_ evolution using different hydroxyl mediators (M). Reaction conditions: 10 mg photocatalyst in 2 ml 1 vol% water-mediator solution with 8 mM C_6_H_11_NO_2_ and 40 mM KOH under 410 nm irradiation (30 mW·cm^−2^) at RT for 0.5 h.


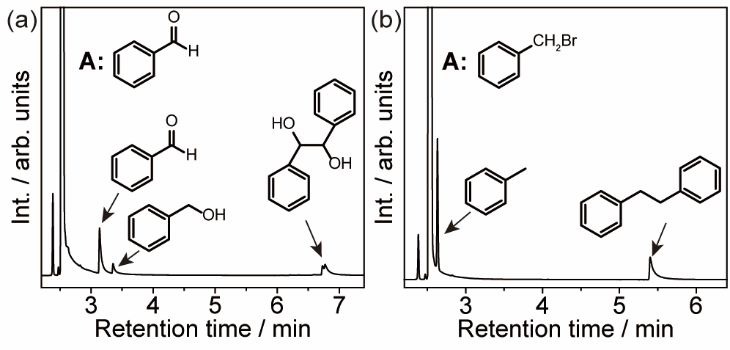


**Figure S14.** GC spectra of photocatalytic H_2_O_2_ evolution using benzaldehyde and benzyl bromide as hydrogen acceptors (A). Reaction conditions: 10 mg photocatalyst in 2 ml 1 vol% water - 1,3-dioxane solution with 8 mM A and 40 mM KOH under 410 nm irradiation (30 mW·cm^−2^) at RT for 0.5 h.

The UV-vis spectra for colorimetric titration of photogenerated H_2_O_2_ at given irradiation time intervals are shown in Figs. S15a and S15b. The photographs of aliquots from the centrifuged reaction suspension added into chromogenic agent are shown in Fig. S15c.


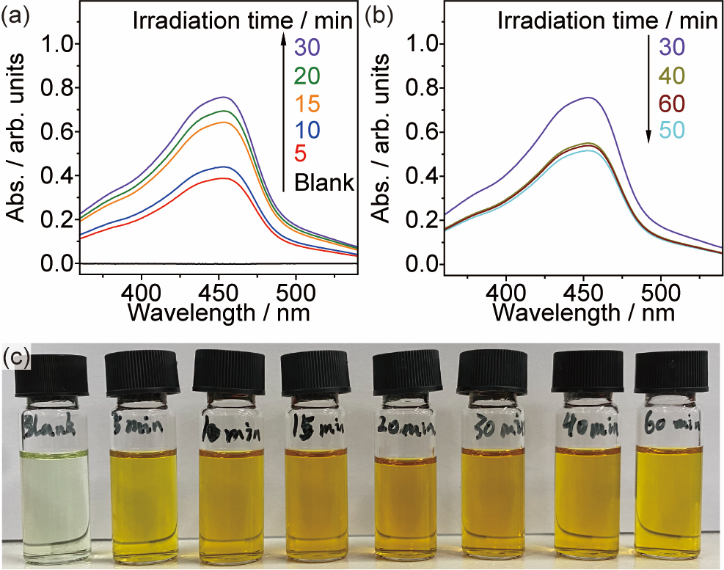


**Figure S15.** Colorimetric titration of photogenerated H_2_O_2_. (a) and (b) UV-vis spectra recorded at given irradiation time intervals. (c) Photographs of the aliquots from the centrifuged reaction suspension added into chromogenic agent.

To identify the presence of radicals in the inert solvent system, a series of control measurements were initially performed using the dioxane solvent without catalyst, as shown in Fig. S14. These results evidence the presence of CdS nanosheet as the key to generate new radical species. The corresponding EPR spectra of CdS nanospheres and nanorods are shown in Fig. S16. Dashed lines correspond to simulations.


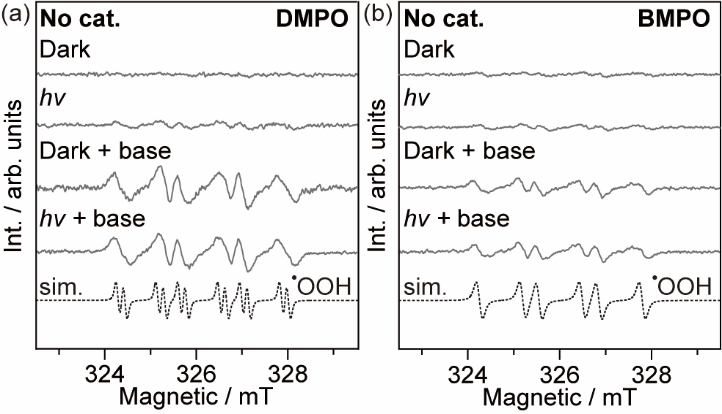


**Figure S16.** CW X-band EPR spectra (T = 298 K) of 1,4-dioxane solution in the absence of photocatalyst under various conditions using (a) DMPO and (b) BMPO as spin trap.


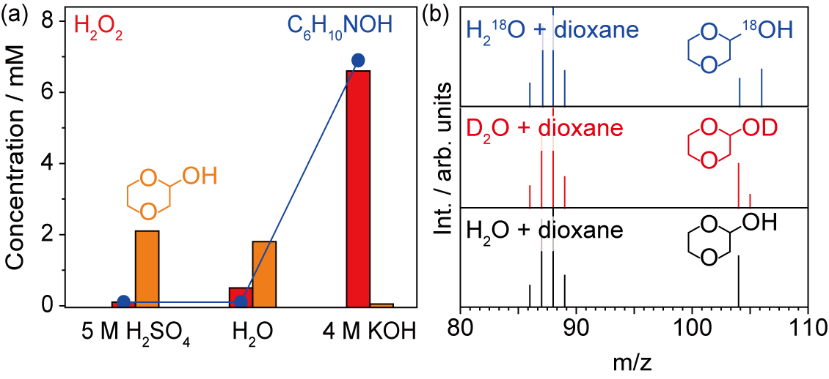


**Figure S17.** (a) The catalytic performance of CdS nanosheets in acidic, neutral, and basic medium. Reaction conditions: 10 mg photocatalyst in 2 ml 1 vol% water-dioxane solution with 8 mM C_6_H_11_NO_2_ and 20 µL solution (5 M H_2_SO_4_, H_2_O, or 4 M KOH) under 410 nm irradiation (30 mW·cm^−2^) at RT for 0.5 h. (b) MS analysis of generated 1,4-dioxane-2-ol during irradiation of CdS nanosheets in acidic media using H_2_O, D_2_O, and H_2_^18^O.


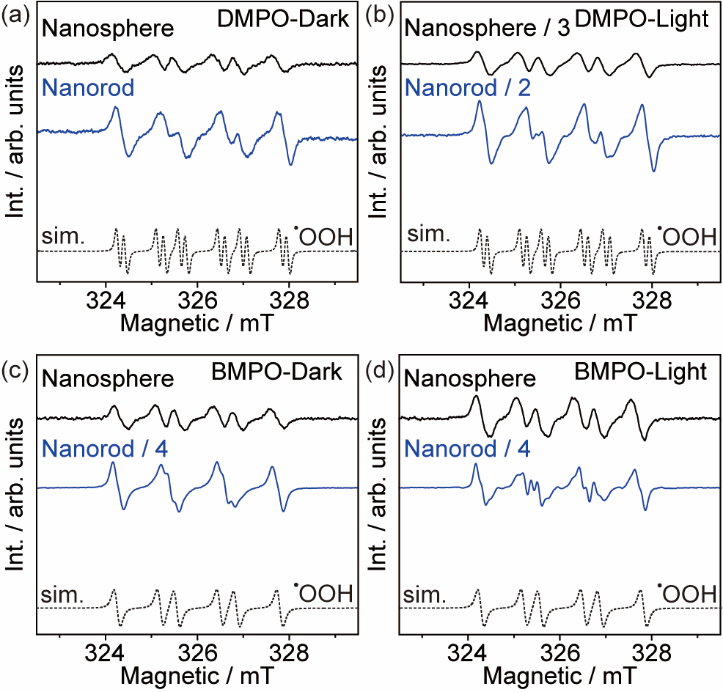


**Figure S18.** CW X-band EPR spectra (T = 298 K) of the photocatalyst-dioxane suspension probed using DMPO and BMPO spin traps. Reaction conditions: 10 mg catalyst in 0.5 mL of 1 vol% 4 M KOH-dioxane with 20 mM spin trap under deaerated conditions, irradiated by a 410 nm LED at RT for 1 min.

An 80 mM of 2,2,6,6-tetramethylpiperidinyloxy (TEMPO, retention time, rt = 3.759 min) was added into the reaction suspension to capture the photogenerated surface adsorbed H atoms (H_ads_) and •OH radicals by GC. Signals were observed for the trapped •OH species (rt = 3.009 min), the trapped H species (rt = 3.660 min) and unreacted TEMPO, respectively.^[35]^ The production of C_6_H_10_NOH and the conversion of C_6_H_11_NO_2_ decreased upon the addition of TEMPO, indicating that Hads was captured by TEMPO, thus slowing down the reduction of C_6_H_11_NO_2_.


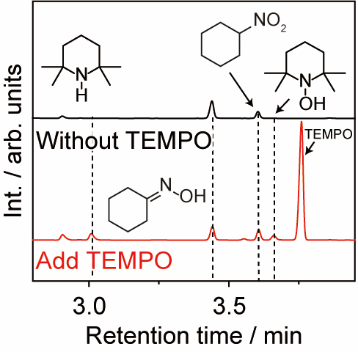


**Figure S19.** GC spectra of the photocatalytic system with/without TEMPO after 0.5 h of irradiation. Reaction conditions: 10 mg catalyst in 2 mL 1 vol % 4 M KOH-dioxane with 40 mM C_6_H_11_NO_2_ under RT and N_2_.


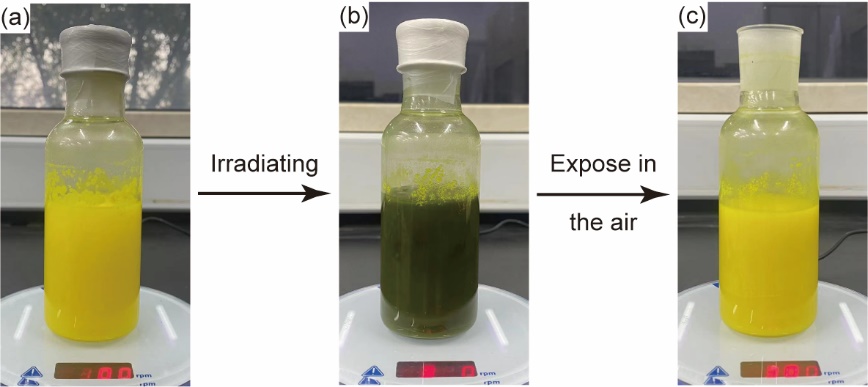


**Figure S20.** Images of CdS nanosheet in 1 vol % water-dioxane (a) before irradiation, (b) post irradiation under deaerated conditions and (c) exposing to the air.

# References

[28] V. Dzimbeg-Malcic, Ž. Barbarić-Mikočević, K. Itrić, *Teh. Vjesn.* **2012**, *18*, 117-124.

[29] S. Stoll, A. Schweiger, *J. Magn. Reson.* **2006**, *178*, 42-55.

[30] A. N. Baga, G. R. A. Johnson, N. B. Nazhat, R. A. Saadalla-Nazhat, *Anal. Chim. Acta* **1988**, *204*, 349-353.

[31] W. J. Haffenden, G. J. Lawson, *Nature* **1966**, *212*, 748-749.

[32] T. He, H. Tang, J. Wu, J. Wang, M. Zhang, C. Lu, H. Huang, J. Zhong, T. Cheng, Y. Liu, Z. Kang, *Nat. Commun.* **2024**, *15*, 7833.

[33] a) S. P. Verevkin, *Thermochim. Acta* **1997**, *307*, 17-25; b) W. V. Steele, R. D. Chirico, A. B. Cowell, S. E. Knipmeyer, A. Nguyen, *J.Chem. Eng. Data* **2002**, *47*, 725-739; c) A. A. Kozyro, G. J. Kabo, V. S. Krouk, M. S. Sheiman, I. A. Yursha, V. V. Simirsky, A. P. Krasulin, V. M. Sevruk, V. I. Gogolinsky, *J.Chem.Eng.Data* **1992**, *24*, 883-895.

[34] a) A. Gopakumar, P. Ren, J. Chen, B. V. Manzolli Rodrigues, H. Y. Vincent Ching, A. Jaworski, S. V. Doorslaer, A. Rokicińska, P. Kuśtrowski, G. Barcaro, S. Monti, A. Slabon, S. Das, *J. Am. Chem. Soc.* **2022**, *144*, 2603-2613; b) Z. Wei, S. Zhao, W. Li, X. Zhao, C. Chen, D. L. Phillips, Y. Zhu, W. Choi, *ACS Catal.* **2022**, *12*, 11436-11443; c) Z. Teng, Q. Zhang, H. Yang, K. Kato, W. Yang, Y.-R. Lu, S. Liu, C. Wang, A. Yamakata, C. Su, B. Liu, T. Ohno, *Nat. Catal.* **2021**, *4*, 374-384; d) X. Zeng, Y. Liu, Y. Kang, Q. Li, Y. Xia, Y. Zhu, H. Hou, M. H. Uddin, T. R. Gengenbach, D. Xia, C. Sun, D. T. McCarthy, A. Deletic, J. Yu, X. Zhang, *ACS Catal.* **2020**, *10*, 3697-3706; e) X. Zhang, H. Su, P. Cui, Y. Cao, Z. Teng, Q. Zhang, Y. Wang, Y. Feng, R. Feng, J. Hou, X. Zhou, P. Ma, H. Hu, K. Wang, C. Wang, L. Gan, Y. Zhao, Q. Liu, T. Zhang, K. Zheng, *Nat. Commun.* **2023**, *14*, 7115; f) A. Gopakumar, P. Ren, J. Chen, B. V. Manzolli Rodrigues, H. Y. Vincent Ching, A. Jaworski, S. V. Doorslaer, A. Rokicińska, P. Kuśtrowski, G. Barcaro, S. Monti, A. Slabon, S. Das, *J. Am. Chem. Soc.* **2022**, *144*, 2603-2613;g) Y. Shiraishi, T. Takii, T. Hagi, S. Mori, Y. Kofuji, Y. Kitagawa, S. Tanaka, S. Ichikawa, T. Hirai, *Nat. Mater.* **2019**, *18*, 985-993; h) S. Wang, Z. Xie, D. Zhu, S. Fu, Y. Wu, H. Yu, C. Lu, P. Zhou, M. Bonn, H. I. Wang, Q. Liao, H. Xu, X. Chen, C. Gu, *Nat. Commun.* **2023**, *14*, 6891; i) Q. Tian, X.-K. Zeng, C. Zhao, L.-Y. Jing, X.-W. Zhang, J. Liu, *Adv. Funct. Mater.* **2023**, *33*, 2213173; j) F. Chen, X. Lv, H. Wang, F. Wen, L. Qu, G. Zheng, Q. Han, *JACS Au* **2024**, *4*, 1219-1228; k) D. Zhang, P. Ren, W. Liu, Y. Li, S. Salli, F. Han, W. Qiao, Y. Liu, Y. Fan, Y. Cui, Y. Shen, E. Richards, X. Wen, M. H. Rummeli, Y. Li, F. Besenbacher, H. Niemantsverdriet, T. Lim, R. Su, *Angew. Chem. Int. Ed.* **2022**, *61*, e202204256; l) P. Ren, T. Zhang, N. Jain, H. Y. V. Ching, A. Jaworski, G. Barcaro, S. Monti, J. Silvestre-Albero, V. Celorrio, L. Chouhan, A. Rokicińska, E. Debroye, P. Kuśtrowski, S. Van Doorslaer, S. Van Aert, S. Bals, S. Das, *J. Am. Chem. Soc.* **2023**, *145*, 16584-16596; m) S. Cao, T.-S. Chan, Y.-R. Lu, X. Shi, B. Fu, Z. Wu, H. Li, K. Liu, S. Alzuabi, P. Cheng, M. Liu, T. Li, X. Chen, L. Piao, *Nano Energy* **2020**, *67*, 104287; n) Z. Zhou, M. Sun, Y. Zhu, P. Li, Y. Zhang, M. Wang, Y. Shen, *Appl. Catal. B* **2023**, *334*, 122862; o) J.-Y. Yue, L.-P. Song, Y.-F. Fan, Z.-X. Pan, P. Yang, Y. Ma, Q. Xu, B. Tang, *Angew. Chem. Int. Ed.* **2023**, *62*, e202309624; p) Y. Zhang, C. Pan, G. Bian, J. Xu, Y. Dong, Y. Zhang, Y. Lou, W. Liu, Y. Zhu, *Nat. Energy* **2023**, *8*, 361-371; q) Q. Liao, Q. Sun, H. Xu, Y. Wang, Y. Xu, Z. Li, J. Hu, D. Wang, H. Li, K. Xi, *Angew. Chem. Int. Ed.* **2023**, *62*, e202310556; r) D. Chen, W. Chen, Y. Wu, L. Wang, X. Wu, H. Xu, L. Chen, *Angew. Chem. Int. Ed.* **2023**, *62*, e202217479; s) C. Wu, Z. Teng, C. Yang, F. Chen, H. B. Yang, L. Wang, H. Xu, B. Liu, G. Zheng, Q. Han, *Adv. Mater.* **2022**, *34*, 2110266; t) P. Das, J. Roeser, A. Thomas, *Angew. Chem. Int. Ed.* **2023**, *62*, e202304349; u) M. Kou, Y. Wang, Y. Xu, L. Ye, Y. Huang, B. Jia, H. Li, J. Ren, Y. Deng, J. Chen, Y. Zhou, K. Lei, L. Wang, W. Liu, H. Huang, T. Ma, *Angew. Chem. Int. Ed.* **2022**, *61*, e202200413; v) L. Chen, L. Wang, Y. Wan, Y. Zhang, Z. Qi, X. Wu, H. Xu, *Adv. Mater.* **2020**, *32*, 1904433; w) Y. Li, Y. Guo, D. Luan, X. Gu, X. W. Lou, *Angew. Chem. Int. Ed.* **2023**, *62*, e202310847.

[35] Q. Wu, J. Ye, W. Qiao, Y. Li, J. W. Niemantsverdriet, E. Richards, F. Pan, R. Su, *Appl. Catal. B* **2021**, *291*, 120118.
